# Serum anion gap is associated with mortality in intensive care unit patients with diastolic heart failure

**DOI:** 10.1038/s41598-023-43928-8

**Published:** 2023-10-04

**Authors:** Hongyu Xu, Jiangling Xia, An Wang, Liwu Zong, Xiaona An, Xiaoling Sun

**Affiliations:** Department of Anesthesiology, Zibo Central Hospital, No. 54 West Communist Youth League Road, Zhangdian District, Zibo, Shandong China

**Keywords:** Cardiology, Diseases, Medical research

## Abstract

Serum anion gap (AG) is closely related to mortality in critically ill patients with several diseases. We aimed to determine the relationship between serum AG levels and 28-day intensive care unit (ICU) mortality in patients with diastolic heart failure (DHF). This cohort study enrolled critically ill patients with DHF from the Medical Information Mart for Intensive Care IV (MIMIC-IV) database. Serum AG levels were calculated using the traditional and albumin-adjusted methods. Multivariate Cox proportional hazards regression and restricted cubic spline curves were used to determine the correlation between serum AG levels and 28-day ICU mortality. We used receiver operating characteristic (ROC) curves and area under the curve (AUC) to compare the ability of traditional and albumin-adjusted AG to predict mortality. Overall, 3290 patients were included. Multivariate analysis showed an association of high levels of traditional (hazard ratio [HR], 1.48; 95% confidence interval [CI], 1.1–1.98, *p* = 0.009) and albumin-adjusted AG (HR, 1.36; 95% CI, 1.02–1.79, *p* = 0.033) with higher risk of 28-day ICU mortality. Restricted cubic spline curves indicated a linear relationship between AG level and 28-day ICU mortality. Comparison of the ROC curves revealed that albumin-adjusted AG had a greater ability to predict 28-day ICU mortality compared with traditional AG (AUCs of 0.569 [95% CI, 0.536–0.601] and 0.619 [95% CI, 0.588–0.649], respectively). In ICU patients with DHF, higher levels of traditional and albumin-adjusted AG were associated with higher 28-day ICU mortality. Albumin-adjusted AG exhibited greater predictive ability for mortality compared with traditional AG.

## Introduction

Diastolic heart failure (DHF), one of two syndromes of heart failure, is common in clinical practice^1^. Approximately half of patients with heart failure seen by clinicians occur DHF^2^. The individuals are more likely to be women, elderly, often have high blood pressure, and associate ventricular hypertrophy^3^. One requirement of the diagnosis of DHF is that the left ventricular ejection fraction (EF) is normal or only slightly reduced (> 50%)^4^. Unfortunately, the seemingly normal EF may lead a part of patients with DHF to be underestimated which hinders timely treatment by the clinicians. One previous study has showed that the mortality of patients with DHF is four times than those without heart failure after matching for age and sex^5^. Therefore, more indicators and biomarkers are needed to explore the prognostic factors of patients with DHF, especially those in ICU, to guide clinicians to pay more attention and take early treatment for these patients.

Serum anion gap (AG) levels can be calculated from electrolytes in the blood using a laboratory test. It is an inexpensive and effective biochemical marker most commonly used in the differential diagnosis of acid–base disorders^6^. Acid–base disorder is also a common complication in critically ill patients with DHF. Two methods are used to calculate serum AG: the traditional method and the albumin-adjusted method^7^. Traditional AG can be influenced by variations in serum albumin concentration^8^, especially hypoalbuminemia. Albumin-adjusted AG, calculated after adjusting for serum albumin, may yield a measurement with greater specificity. A previous study demonstrated that higher levels of albumin-adjusted AG are associated with an increased risk of mortality in patients with early kidney disease^7^. Meanwhile, many recent studies have revealed that serum AG levels may be closely related to mortality in critically ill patients with non-traumatic subarachnoid hemorrhage^9^, acute myocardial infarction^10^, cerebral infarction^11^, disseminated intravascular coagulation^12^, acute pancreatitis^13^, acute kidney injury^14^, and acute ischemic stroke^15^. Another research has confirmed that traditional AG can effectively predict the 30- and 90-day all-cause mortalities of critically ill patients with congestive heart failure (CHF)^16^. However, the effect of serum AG level on mortality in critically ill patients with DHF have been poorly explored. Therefore, the present study aimed to clarify the correlations of traditional and albumin-adjusted AG with 28-day ICU mortality in these individuals.

## Results

### Baseline characteristics of patients

**Figure 1:**
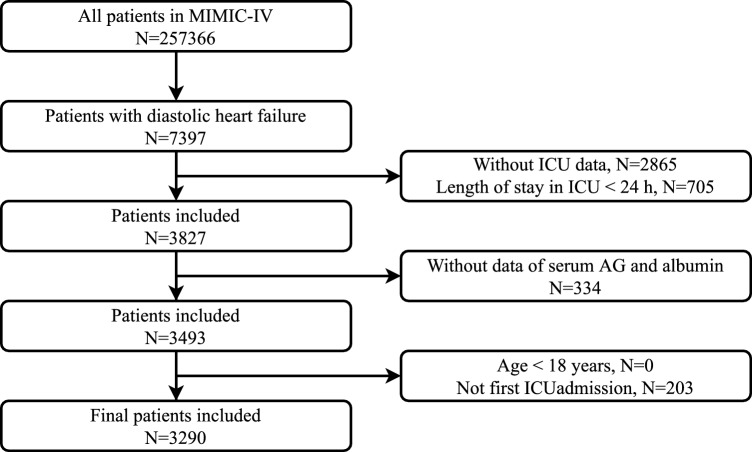
Flowchart of study patients.

A total of 3290 patients were included in this retrospective study (Fig. 1). The baseline characteristics of the traditional and albumin-adjusted AG groups are presented in Tables 1 and 2, respectively. Among the patients, there were 1515 men and 1775 women, and the median age was high, 75.5 ± 12.6 years. Of these, 72.3% were white and 7.5% received RRT.

**Table 1:** Baseline characteristics of the patients with DHF grouped by traditional AG.

Characteristics	Traditional AG (mEq/L)				*p* value
	Over all	< 12	≥ 12, < 15	≥ 15	
	(n = 3290)	(n = 859)	(n = 1490)	(n = 941)	
Age, years	75.5 ± 12.6	75.4 ± 12.7	75.4 ± 12.5	75.7 ± 12.7	0.836
Gender, n (%)					0.399
Male	1515 (46.0)	397 (46.2)	545 (47.4)	573 (44.7)	
Female	1775 (54.0)	462 (53.8)	604 (52.6)	709 (55.3)	
Ethnicity, n (%)					0.177
Black	429 (13.0)	108 (12.6)	146 (12.7)	175 (13.7)	
White	2378 (72.3)	635 (73.9)	846 (73.6)	897 (70)	
Other	483 (14.7)	116 (13.5)	157 (13.7)	210 (16.4)	
Marital status, n (%)					0.034
Married	1525 (46.4)	399 (46.4)	566 (49.3)	560 (43.7)	
Single	696 (21.2)	192 (22.4)	220 (19.1)	284 (22.2)	
Divorced	305 (9.3)	63 (7.3)	109 (9.5)	133 (10.4)	
Widowed	764 (23.2)	205 (23.9)	254 (22.1)	305 (23.8)	
Weight, kg	83.8 ± 27.1	83.2 ± 28.3	83.7 ± 26.0	84.4 ± 27.3	0.619
RRT, n (%)	247 (7.5)	36 (4.2)	66 (5.7)	145 (11.3)	< 0.001
Laboratory tests					
WBC, 10^9/L	10.4 (7.7, 14.1)	10.2 (7.5, 13.7)	10.6 (7.8, 14.2)	10.6 (7.8, 14.2)	0.081
Platelets, 10^9/L	193.5 (144.0, 259.5)	187.5 (136.2, 254.8)	197.5 (145.5, 271.5)	196.2 (147.5, 256.5)	0.059
Albumin, g/dL	3.0 ± 0.7	2.8 ± 0.6	3.0 ± 0.7	3.1 ± 0.7	< 0.001
BNU, mg/dL	28.0 (18.5, 45.9)	24.5 (17.0, 38.0)	26.5 (17.5, 42.5)	35.0 (22.5, 55.0)	< 0.001
Creatinine, mg/dL	1.3 (0.9, 2.1)	1.1 (0.8, 1.6)	1.2 (0.9, 1.9)	1.6 (1.1, 2.8)	< 0.001
Bicarbonate, mEq/L	25.8 ± 5.5	28.6 ± 5.6	26.4 ± 4.7	23.3 ± 4.9	< 0.001
Calcium, mg/dL	8.4 ± 0.9	8.2 ± 0.9	8.4 ± 0.9	8.5 ± 0.9	< 0.001
Chloride, mEq/L	102.5 ± 6.4	104.1 ± 6.4	103.0 ± 6.2	101.0 ± 6.4	< 0.001
Sodium, mEq/L	138.3 ± 4.9	138.8 ± 4.6	138.6 ± 4.7	137.8 ± 5.2	< 0.001
Potassium, mEq/L	4.2 ± 0.7	4.0 ± 0.6	4.1 ± 0.6	4.3 ± 0.8	< 0.001
Lactate, mmol/L	1.5 (1.1, 2.2)	1.5 (1.1, 2.0)	1.5 (1.1, 2.1)	1.6 (1.1, 2.3)	0.001
Glucose, mmol/L	8.1 ± 2.8	7.7 ± 2.5	8.1 ± 2.7	8.3 ± 3.0	< 0.001
PH	7.4 ± 0.1	7.4 ± 0.1	7.4 ± 0.1	7.4 ± 0.1	< 0.001
PO_2_,	129.5 (83.0, 226.0)	121.0 (79.0, 218.5)	139.5 (84.0, 234.5)	126.5 (82.0, 217.0)	0.008
PCO_2_,	44.4 ± 12.4	46.5 ± 14.3	44.4 ± 11.8	43.0 ± 11.3	< 0.001
Vital signs					
Heart rate, beats/min	83.4 ± 15.5	83.3 ± 15.5	83.6 ± 15.5	83.4 ± 15.6	0.918
SBP, mmHg	119.0 ± 17.2	118.1 ± 15.8	119.1 ± 17.4	119.4 ± 17.9	0.192
DBP, mmHg	59.1 ± 10.8	58.7 ± 10.3	59.3 ± 10.3	59.1 ± 11.5	0.516
MBP, mmHg	75.0 ± 10.5	74.4 ± 10.2	75.3 ± 10.1	75.2 ± 11.1	0.164
Respiratory rate, beats/min	19.7 ± 3.8	19.5 ± 3.7	19.7 ± 3.8	19.9 ± 3.7	0.037
Temperature, °C	36.8 ± 0.5	36.8 ± 0.5	36.8 ± 0.5	36.7 ± 0.5	0.5
SpO_2_, %	96.7 ± 2.2	96.7 ± 2.2	96.7 ± 2.2	96.6 ± 2.1	0.536
UO first 24 h, L	2.1 (1.1, 3.8)	2.1 (1.1, 3.8)	2.1 (1.1, 3.8)	2.0 (1.0, 3.9)	0.481
Comorbidity, n (%)					
Hypertension	427 (13.0)	88 (10.2)	157 (13.7)	182 (14.2)	0.02
Chronic pulmonary disease	923 (28.1)	274 (31.9)	322 (28)	327 (25.5)	0.005
Renal disease	988 (30.0)	169 (19.7)	310 (27)	509 (39.7)	< 0.001
Liver disease	53 (1.6)	15 (1.7)	20 (1.7)	18 (1.4)	0.753
Diabetes	822 (25.0)	188 (21.9)	289 (25.2)	345 (26.9)	0.031
Malignant cancer	249 (7.6)	84 (9.8)	81 (7)	84 (6.6)	0.016
Myocardial infarct	219 (6.7)	51 (5.9)	73 (6.4)	95 (7.4)	0.357
Vasopressor drugs, n (%)					
Dopamine	174 (5.3)	30 (3.5)	65 (5.7)	79 (6.2)	0.02
Dobutamine	65 (2.0)	12 (1.4)	17 (1.5)	36 (2.8)	0.023
Epinephrine	172 (5.2)	30 (3.5)	71 (6.2)	71 (5.5)	0.023
Norepinephrine	661 (20.1)	171 (19.9)	216 (18.8)	274 (21.4)	0.283
Phenylephrine	797 (24.2)	200 (23.3)	297 (25.8)	300 (23.4)	0.281
Score systems					
SOFA	5.0 (3.0, 7.0)	5.0 (3.0, 7.0)	5.0 (3.0, 7.0)	5.0 (3.0, 8.0)	< 0.001
SAPS II	40.5 ± 12.8	39.2 ± 11.6	39.8 ± 12.7	42.1 ± 13.3	< 0.001
APS III	49.0 (38.0, 64.0)	47.0 (37.0, 62.0)	48.0 (37.0, 63.0)	51.0 (40.0, 67.0)	< 0.001
Outcomes					
ICU length of stay, days	2.5 (1.6, 4.5)	2.7 (1.6, 4.8)	2.5 (1.5, 4.4)	2.5 (1.6, 4.4)	0.246
28-day ICU mortality, n (%)	393 (11.9)	82 (9.5)	131 (11.4)	180 (14)	0.006
In-hospital mortality, n (%)	643 (19.5)	151 (17.6)	206 (17.9)	286 (22.3)	0.006

Data were mean ± SD or median (IQR) for skewed variables or numbers (proportions) for categorical variables.

*AG* anion gap; *UO* urine output; *RRT* renal replacement therapy; *WBC* white blood cells; *BNU* blood urea nitrogen; *PO*_*2*_ partial oxygen pressure; *PCO*_*2*_ partial pressure of carbon dioxide; *SBP* systolic blood pressure; *DBP* diastolic blood pressure; *MBP* mean blood pressure; *SpO*_*2*_ saturation of peripheral oxygen; *SOFA* sequential organ failure assessment score; *SAPS II* simplified acute physiology score II; *APS III* acute physiology score III; *ICU* intensive care unit.

**Table 2:** Baseline characteristics of the patients with DHF grouped by albumin-adjusted AG.

Characteristics	Albumin-adjusted AG (mEq/L)				*p* value
	Over all	< 14.5	≥ 14.5, < 17.5	≥ 17.5	
	(n = 3290)	(n = 1038)	(n = 1119)	(n = 1133)	
Age, years	75.5 ± 12.6	75.7 ± 12.6	75.5 ± 12.7	75.3 ± 12.7	0.678
Gender, n (%)					0.57
Male	1515 (46.0)	489 (47.1)	502 (44.9)	524 (46.2)	
Female	1775 (54.0)	549 (52.9)	617 (55.1)	609 (53.8)	
Ethnicity, n (%)					< 0.001
Black	429 (13.0)	119 (11.5)	136 (12.2)	174 (15.4)	
White	2378 (72.3)	792 (76.3)	814 (72.7)	772 (68.1)	
Other	483 (14.7)	127 (12.2)	169 (15.1)	187 (16.5)	
Marital status, n (%)					0.111
Married	1525 (46.4)	498 (48)	515 (46)	512 (45.2)	
Single	696 (21.2)	206 (19.8)	239 (21.4)	251 (22.2)	
Divorced	305 (9.3)	78 (7.5)	105 (9.4)	122 (10.8)	
Widowed	764 (23.2)	256 (24.7)	260 (23.2)	248 (21.9)	
Weight, kg	83.8 ± 27.1	84.5 ± 28.5	84.0 ± 26.1	83.1 ± 26.7	0.478
RRT, n (%)	247 (7.5)	33 (3.2)	53 (4.7)	161 (14.2)	< 0.001
Laboratory tests					
WBC, 10^9/L	10.4 (7.7, 14.1)	10.2 (7.5, 13.3)	10.4 (7.8, 14.1)	10.9 (7.9, 14.8)	0.001
Platelets, 10^9/L	193.5 (144.0, 259.5)	183.2 (139.6, 249.9)	200.0 (147.8, 267.0)	199.5 (145.0, 267.0)	0.002
Albumin, g/dL	3.0 ± 0.7	3.2 ± 0.7	3.0 ± 0.7	2.8 ± 0.6	< 0.001
BNU, mg/dL	28.0 (18.5, 45.9)	23.0 (16.5, 35.0)	27.0 (18.5, 42.0)	38.5 (24.0, 59.5)	< 0.001
Creatinine, mg/dL	1.3 (0.9, 2.1)	1.1 (0.8, 1.4)	1.2 (0.9, 1.9)	1.9 (1.1, 3.1)	< 0.001
Bicarbonate, mEq/L	25.8 ± 5.5	29.1 ± 5.1	26.0 ± 4.3	22.5 ± 4.9	< 0.001
Calcium, mg/dL	8.4 ± 0.9	8.6 ± 0.8	8.4 ± 0.9	8.3 ± 0.9	< 0.001
Chloride, mEq/L	102.5 ± 6.4	102.9 ± 6.0	102.9 ± 6.1	101.7 ± 7.0	< 0.001
Sodium, mEq/L	138.3 ± 4.9	138.9 ± 4.5	138.4 ± 4.7	137.8 ± 5.4	< 0.001
Potassium, mEq/L	4.2 ± 0.7	4.0 ± 0.6	4.1 ± 0.6	4.3 ± 0.9	< 0.001
Lactate, mmol/L	1.5 (1.1, 2.2)	1.5 (1.1, 2.0)	1.5 (1.1, 2.1)	1.6 (1.1, 2.4)	< 0.001
Glucose, mmol/L	8.1 ± 2.8	7.8 ± 2.5	8.1 ± 2.7	8.3 ± 3.1	< 0.001
PH	7.4 ± 0.1	7.4 ± 0.1	7.4 ± 0.1	7.4 ± 0.1	< 0.001
PO_2_	129.5 (83.0, 226.0)	141.5 (83.5, 244.8)	130.5 (83.0, 229.5)	120.5 (81.0, 198.0)	< 0.001
PCO_2_	44.4 ± 12.4	47.2 ± 14.2	43.9 ± 11.3	42.3 ± 11.1	< 0.001
Vital signs					
Heart rate, beats/min	83.4 ± 15.5	82.6 ± 14.6	82.9 ± 15.4	84.8 ± 16.3	0.002
SBP, mmHg	119.0 ± 17.2	119.4 ± 16.0	118.8 ± 17.4	118.7 ± 18.1	0.644
DBP, mmHg	59.1 ± 10.8	59.1 ± 9.9	59.1 ± 10.7	59.0 ± 11.5	0.96
MBP, mmHg	75.0 ± 10.5	75.4 ± 9.8	75.0 ± 10.6	74.7 ± 11.2	0.377
Respiratory rate, beats/min	19.7 ± 3.8	19.3 ± 3.7	19.7 ± 3.7	20.1 ± 3.8	< 0.001
Temperature, °C	36.8 ± 0.5	36.8 ± 0.5	36.8 ± 0.5	36.7 ± 0.6	0.149
SpO_2_, %	96.7 ± 2.2	96.6 ± 2.2	96.7 ± 2.1	96.7 ± 2.3	0.861
UO first 24 h, L	2.1 (1.1, 3.8)	2.1 (1.1, 3.8)	2.1 (1.1, 3.8)	2.0 (1.0, 3.9)	0.501
Comorbidity, n (%)					
Hypertension	427 (13.0)	117 (11.3)	156 (13.9)	154 (13.6)	0.137
Chronic pulmonary disease	923 (28.1)	331 (31.9)	317 (28.3)	275 (24.3)	< 0.001
Renal disease	988 (30.0)	198 (19.1)	306 (27.3)	484 (42.7)	< 0.001
Liver disease	53 (1.6)	13 (1.3)	19 (1.7)	21 (1.9)	0.518
Diabetes	822 (25.0)	256 (24.7)	265 (23.7)	301 (26.6)	0.275
Malignant cancer	249 (7.6)	81 (7.8)	88 (7.9)	80 (7.1)	0.726
Myocardial infarct	219 (6.7)	65 (6.3)	78 (7)	76 (6.7)	0.802
Vasopressor drugs, n (%)					
Dopamine	174 (5.3)	33 (3.2)	64 (5.7)	77 (6.8)	< 0.001
Dobutamine	65 (2.0)	14 (1.3)	16 (1.4)	35 (3.1)	0.004
Epinephrine	172 (5.2)	56 (5.4)	57 (5.1)	59 (5.2)	0.951
Norepinephrine	661 (20.1)	147 (14.2)	210 (18.8)	304 (26.8)	< 0.001
Phenylephrine	797 (24.2)	276 (26.6)	252 (22.5)	269 (23.7)	0.079
Score systems					
SOFA	5.0 (3.0, 7.0)	5.0 (3.0, 7.0)	5.0 (3.0, 7.0)	6.0 (4.0, 9.0)	< 0.001
SAPS II	40.5 ± 12.8	37.8 ± 11.6	39.6 ± 11.8	44.0 ± 13.9	< 0.001
APS III	49.0 (38.0, 64.0)	44.0 (36.0, 58.0)	48.0 (38.0, 61.0)	55.0 (43.0, 73.0)	< 0.001
Outcomes					
ICU length of stay, days	2.5 (1.6, 4.5)	2.4 (1.4, 4.2)	2.6 (1.6, 4.4)	2.7 (1.7, 5.1)	< 0.001
28-day ICU mortality, n (%)	393 (11.9)	89 (8.6)	106 (9.5)	198 (17.5)	< 0.001
In-hospital mortality, n (%)	643 (19.5)	143 (13.8)	185 (16.5)	315 (27.8)	< 0.001

Data were mean ± SD or median (IQR) for skewed variables or numbers (proportions) for categorical variables.

*AG* anion gap; *UO* urine output; *RRT* renal replacement therapy; *WBC* white blood cells; *BNU* blood urea nitrogen; *PO*_*2*_ partial oxygen pressure; *PCO*_*2*_ partial pressure of carbon dioxide; *SBP* systolic blood pressure; *DBP* diastolic blood pressure; *MBP* mean blood pressure; *SpO*_*2*_ saturation of peripheral oxygen; *SOFA* sequential organ failure assessment score; *SAPS II* simplified acute physiology score II; *APS III* acute physiology score III; *ICU* intensive care unit.

In the traditional AG group, patients with higher AG levels had higher levels of several laboratory parameters, such as albumin, BUN, creatinine, calcium, potassium, lactate, and glucose. They also had more comorbidities, such as hypertension, diabetes, and renal disease, and received more vasopressor treatments with dopamine and dobutamine. These patients received more RRT and had higher SAPS II and APS III and mortality rates.

Similarly, in the albumin-adjusted AG group, patients with higher AG levels had higher WBC, and BUN, creatinine, potassium, and glucose levels. Interestingly, these patients had lower calcium levels. Their heart and respiratory rates were higher. They had a higher incidence of renal disease, lower incidence of chronic pulmonary disease, and received more vasopressor treatment with dopamine, dobutamine, and norepinephrine. They received RRT more frequently, and their SOFA score, SAPS II, and APS III and mortality rates were higher.

### Association between AGs and 28-day ICU mortality

In the univariate analysis, both traditional and albumin-adjusted AG were associated with an increased risk of 28-day ICU mortality in patients with DHF when used as continuous variables, the hazard ratios (HRs) were 1.05 (95% confidence interval [CI], 1.03–1.07; *p* < 0.001), 1.05 (95% CI, 1.03–1.06; *p* < 0.001), respectively. As shown in Table 3, AGs measured using the two methods were used as categorical variables, with the group with the lowest AG level as the reference group. The group with high levels of traditional AG showed significantly increased 28-day ICU mortality (HR, 1.68; 95% CI, 1.3–2.19; *p* < 0.001). The same effect was replicated in the group with high levels of albumin-adjusted AG (HR, 1.57; 95% CI, 1.22–2.02; *p* < 0.001). In contrast, the groups with moderate levels of traditional and albumin-adjusted AG both showed no significant association with 28-day ICU mortality. The variables of age; WBC count; BUN, creatinine, and lactate levels; pH; PO_2_; SBP; DBP; MBP; respiratory rate; temperature; SpO_2_; presence of hypertension, liver disease, and malignant cancer; use of dobutamine, phenylephrine, and norepinephrine; SOFA score; SAPS II; and APS III were associated with 28-day ICU mortality.

**Table 3:** Results of univariate analysis for 28-day ICU mortality.

Variable	HR (95%CI)	*P* value
Traditional AG, mEq/L	1.05 (1.03,1.07)	< 0.001
< 12	ref	
≥ 12, < 15	1.3 (0.98,1.71)	0.064
≥ 15	1.68 (1.3,2.19)	< 0.001
Albumin-adjusted AG, mEq/L	1.05 (1.03,1.06)	< 0.001
< 14.5	ref	
≥ 14.5, < 17.5	1.02 (0.77,1.36)	0.875
≥ 17.5	1.57 (1.22,2.02)	< 0.001
Age, years	1.04 (1.03,1.05)	< 0.001
WBC, 10^9/L	1.01 (1,1.02)	0.003
BNU, mg/dL	1.01 (1.006,1.012)	< 0.001
Creatinine, mg/dL	1.06 (1.01,1.12)	0.027
Bicarbonate, mEq/L	0.96 (0.95,0.98)	< 0.001
Potassium, mEq/L	1.25 (1.11,1.4)	< 0.001
Lactate, mmol/L	1.2 (1.15,1.26)	< 0.001
PH	0.12 (0.03,0.44)	0.001
PO_2_	0.997 (0.995,0.998)	< 0.001
SBP, mmHg	0.98 (0.97,0.99)	< 0.001
DBP, mmHg	0.99 (0.98,1)	0.012
MBP, mmHg	0.97 (0.96,0.98)	< 0.001
Respiratory rate, beats/min	1.04 (1.01,1.07)	0.002
Temperature, °C	0.71 (0.61,0.82)	< 0.001
SpO_2_, %	0.94 (0.9,0.97)	< 0.001
Hypertension, n (%)	0.63 (0.42,0.95)	0.028
Liver disease, n (%)	1.79 (1,3.18)	0.048
Malignant cancer, n (%)	2.07 (1.54,2.78)	< 0.001
Dobutamine, n (%)	1.96 (1.35,2.83)	< 0.001
Norepinephrine, n (%)	1.34 (1.09,1.65)	0.005
Phenylephrine, n (%)	0.74 (0.6,0.92)	0.008
SOFA	1.08 (1.05,1.11)	< 0.001
SAPS II	1.03 (1.03,1.04)	< 0.001
APS III	1.01 (1.01,1.02)	< 0.001

Data presented are HRs and 95% CIs.

*ICU* intensive care unit; *AG* anion gap; *WBC* white blood cells; *BNU* blood urea nitrogen; *PO*_*2*_ partial oxygen pressure; *SBP* systolic blood pressure; *DBP* diastolic blood pressure; *MBP* mean blood pressure; *SOFA* sequential organ failure assessment score; *SAPS II* simplified acute physiology score II; *APS III* acute physiology score III.

Table 4 shows the adjusted analysis of AGs with 28-day ICU mortality in patients with DHF using multivariate Cox proportional hazards models. In Model I, when used as continuous variables, traditional (HR, 1.05; 95% CI, 1.03–1.07; *p* < 0.001) and albumin-adjusted AG (HR, 1.05; 95% CI, 1.03–1.07; *p* < 0.001) were associated with increased risk of 28-day ICU mortality with adjustments for sex, age, marital status, and ethnicity. When used as categorical variables, the groups with high levels of traditional and albumin-adjusted AG demonstrated a higher risk of 28-day ICU mortality, the adjusted HRs were 1.64 (95% CI, 1.26–2.13; *p* < 0.001) and 1.61 (95% CI, 1.25–2.07; *p* < 0.001), respectively. After adjustment in Model II, no significant effects of traditional and albumin-adjusted AG as continuous variables were observed on 28-day ICU mortality. Similar to Model I, groups with high levels of traditional (HR, 1.48; 95% CI, 1.1–1.98; *p* = 0.009) and albumin-adjusted AG (HR, 1.36; 95% CI, 1.02–1.79; *p* = 0.033) remained significantly associated with an increased risk of 28-day ICU mortality, wherein the groups with the lowest AG levels were considered the reference groups. However, the groups with moderate levels of traditional (HR, 1.31; 95% CI, 0.98–1.74; *p* = 0.066) and albumin-adjusted AG (HR, 1.07; 95% CI, 0.8–1.43; *p* = 0.655) yielded the same results as in Model I, of no significant associations with 28-day ICU mortality.

**Table 4:** Multivariable cox proportional hazard models analysis of AG with 28-day ICU mortality.

Anion Gap	Crude		Model I		Model II	
	HR (95% CI)	*p* value	HR (95% CI)	*p* value	HR (95% CI)	*p* value
Traditional AG, mEq/L	1.05 (1.03–1.07)	< 0.001	1.05 (1.03–1.07)	< 0.001	1.02 (1–1.04)	0.024
< 12	Ref		Ref		Ref	
≥ 12, < 15	1.3 (0.98–1.71)	0.064	1.31 (0.99–1.72)	0.059	1.31 (0.98–1.74)	0.066
≥ 15	1.68 (1.3–2.19)	< 0.001	1.64 (1.26–2.13)	< 0.001	1.48 (1.1–1.98)	0.009
*p* for trend		< 0.001		< 0.001		0.01
Albumin-adjusted AG, mEq/L	1.05 (1.03,1.06)	< 0.001	1.05 (1.03–1.07)	< 0.001	1.02 (1–1.04)	0.055
< 14.5	ref		Ref		Ref	
≥ 14.5, < 17.5	1.02 (0.77–1.36)	0.875	1.03 (0.77–1.36)	0.862	1.07 (0.8–1.43)	0.655
≥ 17.5	1.57 (1.22–2.02)	< 0.001	1.61 (1.25–2.07)	< 0.001	1.36 (1.02–1.79)	0.033
*p* for trend		< 0.001		< 0.001		0.025

Data presented are ORs and 95% CIs.

*AG* anion gap.

Model I adjusted for gender, age, marital status, ethnicity.

Model II adjusted for model I + weight, albumin (for traditional AG), BUN, lactate, PH, PO_2_, PCO_2,_ HR, SBP, RR, temperature, SpO_2_, chronic pulmonary disease, renal disease, liver disease, diabetes, hypertension, malignant cancer, dopamine, dobutamine, phenylephrine, RRT, SAPS II, APS III.

**Figure 2:**
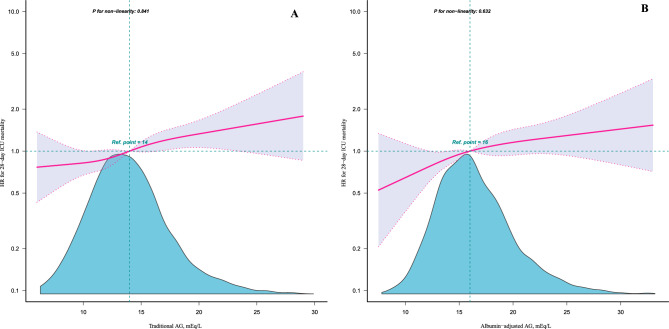
Relationship between serum AGs measured in two methods and the risk of 28-day ICU mortality. (**A**) Traditional AG, (**B**) albumin-adjusted AG. The solid pink lines represent the smooth curves fit between variables. Purple bands depict the 95% confidence intervals. Blue areas show sample size distribution. Data were adjusted for gender, age, marital status, ethnicity, weight, albumin (only for traditional AG), BUN, lactate, PH, PO_2_, PCO_2_, heart rate, SBP, respiratory rate, temperature, SpO_2_, chronic pulmonary disease, renal disease, liver disease, diabetes, hypertension, malignant cancer, dopamine, dobutamine, phenylephrine, RRT, SAPS II, APS III.

**Figure 3:**
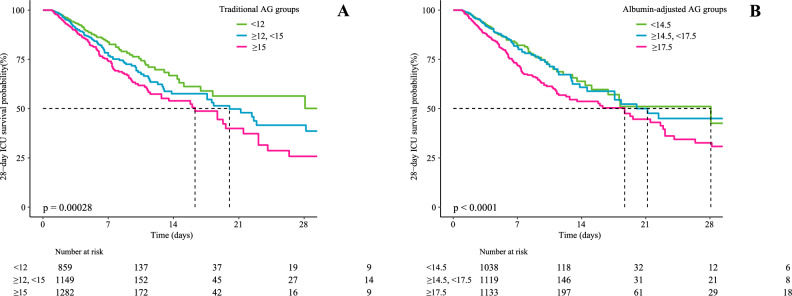
Kaplan–Meier survival curves for patients with DHF in ICU based on (**A**) traditional AG and (**B**) albumin-adjusted AG. x-axis: survival time (days). y-axis: survival probability.

Restricted cubic spline curves indicated a linear relationship between AGs and 28-day ICU mortality after adjusting for confounders, as shown in Fig. 2. The results are similar to those of P for the trends listed in Table 4. The Kaplan–Meier (K–M) survival curve illustrated that patients with elevated levels of traditional and albumin-adjusted AG had decreased survival rates, as shown in Fig. 3 (*p* < 0.0001).

### ROC curve analysis

**Figure 4:**
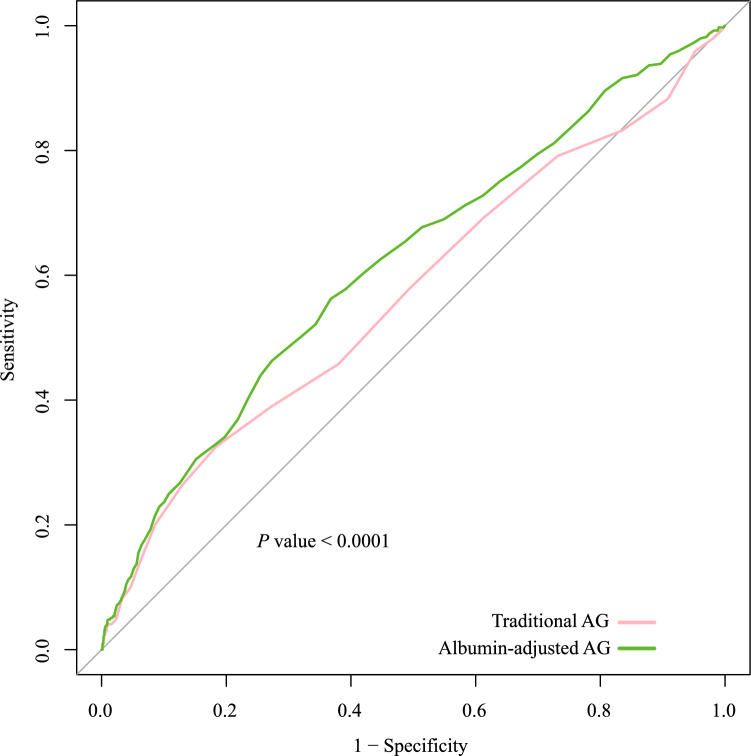
Comparison of receiver operating characteristic curves between traditional AG and albumin-adjusted AG for predicting 28-day ICU mortality.

A comparison of the ROC curves between traditional AG and albumin-adjusted AG revealed a significant difference. The ability of albumin-adjusted AG to predict 28-day ICU mortality was higher than that of traditional AG (AUCs of 0.569 [95% CI, 0.536–0.601] and 0.619 [95% CI: 0.588–0.649], respectively) (Fig. 4).

### Subgroup analysis

**Figure 5:**
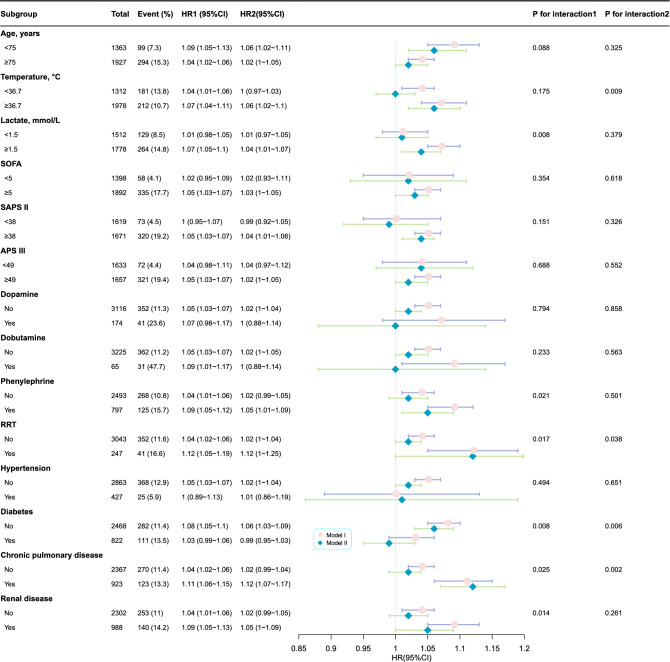
Subgroup analysis of the correlation between traditional AG and 28-day ICU mortality. Adjustment factors included gender, age, marital status, ethnicity, weight, albumin (only for traditional AG), BUN, lactate, PH, PO_2_, PCO_2_, heart rate, SBP, respiratory rate, temperature, SpO_2_, chronic pulmonary disease, renal disease, liver disease, diabetes, hypertension, malignant cancer, dopamine, dobutamine, phenylephrine, RRT, SAPS II, APS III.

Subgroup analysis was applied to evaluate the trend of effect sizes between the traditional AG and 28-day ICU mortality in Models I and II (Fig. 5). There were no significant interactions in most strata of the two models, except for the receipt of RRT (P for interaction in model I = 0.017; P for interaction in model II = 0.038) and the presence of diabetes (P for interaction in model I = 0.008; P for interaction in model II = 0.006) and chronic pulmonary disease (P for interaction in model I = 0.025; P for interaction in model II = 0.002). Additionally, we noted that a few variables such as lactate level (P for interaction = 0.008), phenylephrine use (P for interaction = 0.021), and presence of renal disease (P for interaction = 0.014) showed an interaction between traditional AG and 28-day ICU mortality in Model I. Meanwhile, temperature (P for interaction = 0.009) showed a similar interaction in Model II. Consistent results were observed in Supplementary Tables S4 and S5.

**Figure 6:**
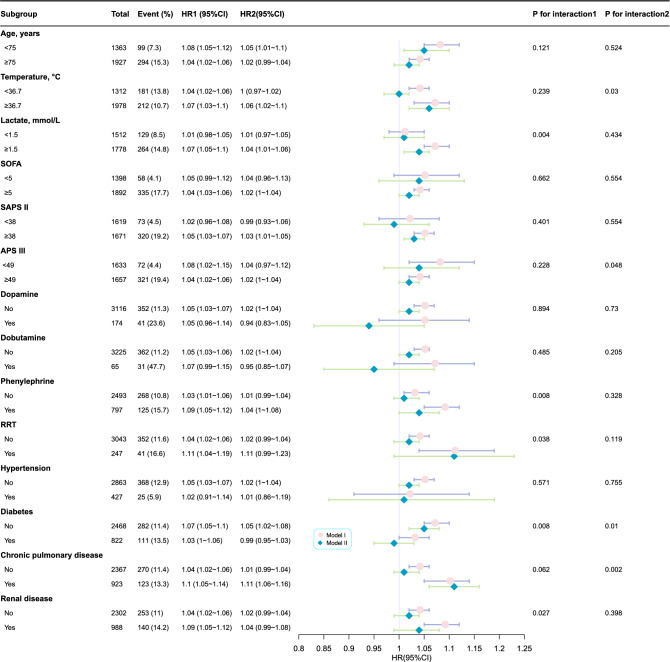
Subgroup analysis of the correlation between albumin-adjusted AG and 28-day ICU mortality. Adjustment factors included gender, age, marital status, ethnicity, weight, albumin (only for traditional AG), BUN, lactate, PH, PO_2_, PCO_2_, heart rate, SBP, respiratory rate, temperature, SpO_2_, chronic pulmonary disease, renal disease, liver disease, diabetes, hypertension, malignant cancer, dopamine, dobutamine, phenylephrine, RRT, SAPS II, APS III.

Similar results were observed between the albumin-adjusted AG and 28-day ICU mortality, as shown in Fig. 6. Only presence of diabetes showed an interaction in the two models (P for interaction in Model I = 0.008; P for interaction in Model II = 0.01). Variables such as lactate level (P for interaction = 0.004), phenylephrine use (P for interaction = 0.008), receipt of RRT (P for interaction = 0.038), and presence of renal disease (P for interaction = 0.027) demonstrated an interaction between albumin-adjusted AG and 28-day ICU mortality in Model I. Additionally, variables such as temperature (P for interaction = 0.03), APS III (P for interaction = 0.048), and presence of chronic pulmonary disease (P for interaction = 0.002) demonstrated a similar interaction. Consistent results are presented in Supplementary Tables S6 and S7.

## Discussion

This study demonstrated the relationship between serum AG levels and 28-day ICU mortality in patients with DHF. Serum AG levels can be calculated using the traditional and albumin-adjusted methods. There was a potential linear trend between AGs measured using the two methods and mortality; higher serum AG levels were associated with an increased risk of 28-day ICU mortality and a shorter survival time. Both traditional and albumin-adjusted AG remained independent predictors of 28-day ICU mortality in critically ill patients with DHF after adjusting for confounders. Moreover, no significant interactions were found between the AGs and 28-day ICU mortality in most subgroups. Specifically, the ROC curves of traditional and albumin-adjusted AG showed a significant difference in mortality prediction. The predictive ability of albumin-adjusted AG was greater than that of traditional AG, with AUCs of 0.619 and 0.569, respectively.

Serum AG is an inexpensive and effective tool that aids in the detection of various acid–base imbalances and differential diagnosis of metabolic acidosis. It can be calculated with serum or plasma electrolytes; serum values are used most frequently, and reflect the differences between the concentrations of unmeasured anions and cations^6^. The average serum AG in healthy individuals measured in mEq/L is 12 ± 4^17^. With the invention of automated analyzers that enabled measurement of electrolytes in large groups of patients, particularly in critically ill patients, the serum AG can be obtained easier and easier. As such, many previous studies have revealed the relationship between serum AG levels and clinical outcomes or predicting prognosis of critically ill patients. One study found that high serum AG levels were a significant risk for ICU and hospital all-cause mortality in patients with non-traumatic SAH^9^. Tang et al.^16^ also measured the association between serum AG and the 30-day and 90-day all-cause mortalities of critically ill patients with CHF and suggested that high AG levels were associated with increased risk mortality. Another retrospective study of critically ill patients with acute ischemic stroke demonstrated there was a potential linear trend between the plasma AG and in-hospital mortality and revealed that the AG was an independent risk factor for in-hospital mortality^15^. Similarly, our findings were consistent with these studies and showed a positive correlation between serum AG and 28-day ICU mortality in critically ill patients with DHF. Most studies used traditional AG to explore the relationship between serum AG levels and clinical outcomes of critically ill patients, however, this study compared the association between traditional AG and albumin-adjusted AG to mortality in critically ill patients.

Most of the serum AG is due to the sum of anionic charges on circulating proteins, of which the most abundant one under normal conditions is albumin^18^. Hence, changes in the concentration of serum albumin can alter serum AG levels, especially in individuals with hypoalbuminemia^6^. Therefore, Serum AG levels should be corrected for serum albumin to obtain an albumin-adjusted AG. Abramowitz et al.^7^ reported that higher serum AG levels, calculated using three methods (traditional AG, albumin-adjusted AG and full AG), were associated with an increased risk of mortality independent of eGFR and albuminuria. Kim et al. indicated that corrected AG may be used to predict mortality in children, and that an elevated level of corrected AG at admission was associated with higher mortality in the pediatric ICU^19^. Similarly, albumin-adjusted AG was used to evaluate its relationship with mortality to reduce bias and increase predictive value in our study. We observed the same trend, enhancing the stability and consistency of the results. Furthermore, there was a significant difference between traditional and albumin-adjusted AG in prediction of 28-day ICU mortality. However, forecasting the risk of mortality by using a single measurement of either traditional or albumin-adjusted AG may not be effective. Therefore, a more accurate evaluation strategy for predicting clinical outcomes should be considered in patients with DHF.

However, the specific mechanism underlying the close correlation between serum AG levels and 28-day ICU mortality in patients with DHF remains unclear. DHF can occur when a ventricle with normal or low end-diastolic volume cannot accept a normal venous return^1^. Its pathophysiology mainly includes impaired left ventricular filling, delayed relaxation, and increased stiffness. As a result, an upward displacement of the diastolic pressure–volume relationship with increased end-diastolic, left atrial, and pulmonary wedge pressure may be generated, leading to symptoms of pulmonary congestion^20^. Signs and symptoms such as breathlessness, coughing, tachypnea, dyspnea on exertion, or paroxysmal nocturnal dyspnea may be present^2^, which may cause respiratory acid–base imbalance. Meanwhile, circulatory disturbances are caused by the heart losing its ability to efficiently pump blood, leading to a decreased perfusion of tissue, hypoxia of cells, and metabolic acid–base imbalance^21^. Ongoing acid–base imbalance can aggravate the severity of the disease and is associated with poor prognosis of the patient^22^. Serum AG can reflect various acid–base imbalances; this is the explanation that we propose to clarify the relationship between serum AG levels and 28-day ICU mortality in patients with DHF.

More recently, DHF has been redefined as HF with normal or preserved EF (HFnEF or HFpEF)^1^. Additionally, Bozkurt et al.^23^ proposed a new and revised classification of HF according to the LVEF and ended a history of inconsistent definitions and classifications of HF in March 2021. Symptomatic HF with LVEF ≥ 50% is defined as HFnEF or HFpEF. Because the information of patients in the MIMIC-IV database was recorded from 2008 to 2019, the previous standard for diagnosing DHF was used. The patients with DHF in our study, whose EF were ≥ 0.50, were similar to individuals with HFnEF or HFpEF. Therefore, the findings of this retrospective study may be applicable to patients with HFnEF and HFpEF.

To our knowledge, this is the first study to show an independent association of traditional and albumin-adjusted AGs with 28-day ICU mortality in patients with DHF admitted to the ICU. However, the present study has some limitations. Firstly, due to the nature of the MIMIC-IV database, the absence of some variables, such as lactate level, pH, PO_2_, and PCO_2_ was close to 35%, and multiple imputations based on five replications were employed to reduce bias. Secondly, the diagnosis of DHF was based on administrative codes. Although the first sequence of diagnosis was used, it is possible that some false associations were caused by misclassifications. Thirdly, although we had performed Multivariate Cox proportional hazard regression models to control the confounding, there would exist some potential confounders that would not be measured in our study. Fourth, this was a single-institution retrospective study, and there may have been a selection bias. Therefore, these results may not apply to all patients with DHF and should be regarded only as of reference and must be further verified. Nevertheless, the independent associations of traditional and albumin-adjusted AG with 28-day ICU mortality remain unclear.

In conclusion, this retrospective observational study showed that higher levels of traditional and albumin-adjusted AG were associated with higher 28-day ICU mortality in patients with DHF. AG measured using both traditional and albumin-adjusted methods could be used as simple and effective tools to predict the 28-day ICU mortality in these patients. Furthermore, the predictive ability for mortality of albumin-adjusted AG was greater than that of traditional AG.

## Methods

### Study design and data source

This retrospective cohort study adhered to the Strengthening the Reporting of Observational Studies in Epidemiology statement^24^. Data were collected from the Medical Information Mart for Intensive Care IV (MIMIC-IV version 1.0) database (https://mimic.mit.edu/)^25^, which contains information on patients at a Tertiary Academic Medical Center from 2008 to 2019. The Beth Israel Deaconess Medical Center (BIDMC, Boston, MA, USA) approved the permission to use the database. All researchers who complete the Collaborative Institutional Training Initiative examination can access it for data extraction purposes. One author of this study had completed the examination and received the permission (certification number 40287955 for Hongyu Xu). All data in the database were de-identified to protect patient privacy. Therefore, informed consent was not required.

### Study population and data extraction

A total of 257,366 individuals were included in the MIMIC-IV database from 2008 to 2019, of whom 50,048 were admitted to the ICU. Among them, 7397 patients with DHF were selected using the ninth version of the International Classification of Diseases (ICD-9) codes 42830,42831,42832, and 42833 and the ICD-10 codes 1503,15030,15031,15032, and 15033. We excluded patients based on the following criteria: (1) patients without ICU data within 24 h of ICU admission; (2) patients with ICU length of stay < 24 h; (3) patients without data of interest such as serum AG and albumin; and (4) patients aged < 18 years. For patients admitted to the ICU more than once, only data from the first ICU admission were included. Thus, 3290 patients were included in this study.

All variables were extracted from the MIMIC-IV database using a structured query language with PostgreSQL tools (version 9.6). The variables of interest and major exposure factors were the first available levels of serum AG and albumin after ICU admission. From the table named “patients” in MIMIC-IV database, we obtained survival information of the primary outcome variable. Other variables included demographics, comorbidities, scoring systems, use of vasopressors, laboratory test results, and vital signs. The demographic variables included sex, age, ethnicity, and marital status. The comorbidities included hypertension, chronic pulmonary disease, renal disease, liver disease, diabetes, malignant cancer, and myocardial infarction. The severity of disease on admission was assessed using the sequential organ failure assessment (SOFA) score, simplified acute physiology score (SAPS) II, and acute physiology score (APS) III. The vasopressors included dopamine, dobutamine, epinephrine, norepinephrine, and phenylephrine. We observed the first 24-h ICU records of patients and calculated the average of multiple results within 24 h after ICU admission to obtain average values of laboratory tests results and vital signs. Laboratory tests included white blood cell (WBC) and platelet counts; blood urea nitrogen (BUN), creatinine, bicarbonate, calcium, chloride, sodium, potassium, lactate, and glucose levels; pH; and partial pressures of oxygen (PO_2_) and carbon dioxide (PCO_2_). Variables such as heart rate, systolic blood pressure (SBP), diastolic blood pressure (DBP), mean blood pressure (MBP), respiratory rate, temperature, saturation of peripheral oxygen (SpO_2_), and urine output during the first 24 h were considered as the vital signs. Weight gain and renal replacement therapy (RRT) were administered. Potential bias was avoided by excluding variables with missing values > 35%. Details of the missing values are presented in Supplementary Table S1.

### Definition of traditional AG and albumin-adjusted AG

Traditional AG was defined by the following equation: traditional AG = [serum sodium(mEq/L) + serum potassium (mEq/L)] − [serum chloride (mEq/L) + serum bicarbonate (mEq/L)]. Albumin-adjusted AG was obtained using the following formula: albumin-adjusted AG = traditional AG + 2.5 × [4 − serum albumin(g/dL)]^6,26^.

### Statistical analysis

Continuous variables were described as means with standard deviations (SD) or medians with interquartile ranges, and categorical variables were presented as frequencies or percentages. The baseline characteristics of the different groups were analyzed using one-way analysis of variance or the Kruskal–Wallis H test for continuous variables and the chi-square test for categorical variables. Multivariate Cox proportional hazards regression models were used to evaluate the relationship between traditional and albumin-adjusted AG and 28-day ICU mortality. Variables that were entered into the regression models as confounders were those based on clinical judgment or those with a change in the effect estimate > 10%. We adjusted for demographic factors such as sex, age, marital status, and ethnicity in Model I. In Model II, we adjusted for factors included in Model I in addition to weight; albumin (only for traditional AG), BUN, and lactate levels; pH; PO_2_; PCO_2_; heart rate; SBP; respiratory rate; temperature; SpO_2_; presence of chronic pulmonary disease, renal disease, liver disease, diabetes, hypertension, and malignant cancer; use of dopamine, dobutamine, and phenylephrine; receipt of RRT; SAPS II; and APS III. Potential multi-collinearity was tested using the variance inflation factor (VIF), with VIF ≥ 2 indicating the presence of multi-collinearity (Supplementary Tables S2 and S3). Due to multicollinearity, we eliminated variables such as bicarbonate, chloride, sodium, potassium, and SOFA score.

To examine the linear associations of traditional and albumin-adjusted AG with 28-day ICU mortality in patients with DHF, we used restricted cubic spline curves based on Cox proportional hazards regression models adjusted for all variables. Traditional and albumin-adjusted AG values were also converted into categorical variables by categorizing into tertiles. Tests for trend were calculated to further explore linearity by entering the median value of traditional and albumin-adjusted AG of each group as continuous variables in the models. We constructed receiver operating characteristic (ROC) curves and calculated AUCs to compare the ability of traditional and albumin-adjusted AG to predict 28-day ICU mortality. Additionally, K-M curves were constructed to visualize the differences in survival between different groups of patients with AGs measured using the two methods. Subgroup analyses were performed using Cox proportional hazards models to identify modifications and interactions. Tests of interaction were performed for all subgroups, followed by the likelihood ratio test. The subgroups were classified based on age (< 75 or ≥ 75 years); temperature (< 36.7 or ≥ 36.7 °C); lactate (< 1.5 or ≥ 1.5 mmol/L); SOFA score (< 5 or ≥ 5 points); SAPS II (< 38 or ≥ 38 points); APS III (< 49 or ≥ 49 points); use of dopamine, dobutamine, and phenylephrine; receipt of RRT; and presence of hypertension, diabetes, chronic pulmonary disease, and renal disease. Multiple imputations, based on five replications and a chained-equation approach method in the R-MI procedure, were used to impute missing data of less than 35%^27^.

The statistical software packages R 3.3.2 (http://www.R-project.org, The R Foundation) and Free Statistics software versions 1.7.1 (Beijing, China) were used for all the analyses. A two-tailed test was performed and statistical significance was set at *p* < 0.05.

### Ethics approval and consent to participate

The MIMIC-IV database was approved by the Massachusetts Institute of Technology (Cambridge, MA) and Beth Israel Deaconess Medical Center (Boston, MA), and consent was obtained for the original data collection.

### Supplementary Information

Supplementary Tables.

## Data Availability

The datasets are available in the physionet (https://physionet.org/content/mimiciv/1.0/).

## Abbreviations

AG: Anion gap
ICU: Intensive care unit
DHF: Diastolic heart failure
MIMIC-IV: Medical information mart for intensive care-IV
K–M: Kaplan–Meier
ROC: Receiver operating characteristic
AUC: Area under the ROC curve
EF: Ejection fraction
ICD: International classification of diseases
SQL: Structured query language
LOS: Length of stay
SOFA: Sequential organ failure assessment
SAPS II: Simplified acute physiology score II
APS III: Acute physiology score III
WBC: White blood cells
BNU: Blood urea nitrogen
PO_2_: Partial oxygen pressure
PCO_2_: Partial pressure of carbon dioxid
SBP: Systolic blood pressure
DBP: Diastolic blood pressure
MBP: Mean blood pressure
SpO_2_: Saturation of peripheral oxygen
UO: Urine output
RRT: Renal replacement therapy
