# Supplementary material for: Serum anion gap is associated with mortality in intensive care unit patients with diastolic heart failure
Source: Sci Rep. 2023 Oct 4;13:16670. doi: 10.1038/s41598-023-43928-8 (PMC10550980; doi:10.1038/s41598-023-43928-8)
Supplement: Supplementary file 1 — Supplementary Tables. [file 41598_2023_43928_MOESM1_ESM.pdf]

Table S1 Details of missing values

Variable	The number of missing values	The percent of missing values (%)
BUN	9	0.27
Calcium	577	17.54
Creatinine	8	0.24
DBP	11	0.33
UO first 24h	98	2.98
Glucose	8	0.24
Heart rate	3	0.09
Lactate	1151	34.98
Marital status	65	1.98
MBP	3	0.09
PCO_2	1150	34.95
PH	1150	34.95
Platelets	14	0.43
PO_2	1150	34.95
Respiratory rate	3	0.09
SBP	11	0.33
SPO_2	4	0.12
Temperature	151	4.59
WBC	15	0.46
Weight	50	1.52

Abbreviations: BNU, blood urea nitrogen; DBP, diastolic blood pressure; UO, urine output; MBP, mean blood pressure; PCO2, partial pressure of carbon dioxide; PO2, partial oxygen pressure; SBP, systolic blood pressure; SpO2, saturation of peripheral oxygen; WBC, white blood cells.

Table S2 Selection of variables for traditional AG

Variables	Change percentage in model I	Change percentage in model II	VIF	colinearity	select
Gender, n (%)	0.5	11.8	1.208	0	Yes
Age, years	-1.1	-29.8	1.27	0	Yes
Ethnicity, n (%)	0	-10	1.109	0	Yes
Marital status, n (%)	6.2	-3.1	1.083	0	No
Weight, kg	-8.4	49	1.182	0	Yes
Platelets, 10^9/L	2.1	2.3	1.184	0	No
WBC, 10^9/L	-0.9	0.4	1.12	0	No
Albumin, g/dL	-0.3	16.4	1.348	0	Yes
BNU, mg/dL	-15.5	-0.5	1.487	0	Yes
Creatinine, mg/dL	-3.7	-0.6	1.89	0	No
Bicarbonate, mEq/L	-27.3	-27.9	6.801	1	Pending
Calcium, mg/dL	0.1	3.1	1.262	0	No
Chloride, mEq/L	11.6	-93.6	7.235	1	Pending
Sodium, mEq/L	-1.4	-117.6	5.358	1	Pending
Potassium, mEq/L	-7.2	38.3	2.462	1	Pending
Lactate, mmol/L	-21.3	-1.2	1.399	0	Yes
Glucose, mmol/L	0.1	-0.2	1.16	0	No
PH	-6.2	18.2	1.419	0	Yes
PO_2	2.1	-31.8	1.283	0	Yes
PCO_2	9.6	-8.4	1.583	0	No
Heart rate, beats/min	-2	1.8	1.303	0	No
SBP, mmHg	0.1	-18.8	2.073	1	Pending
DBP, mmHg	3.1	2.5	2.851	1	Pending
MBP, mmHg	5.1	5.6	3.424	1	Pending
Respiratory rate, beats/min	3.3	-0.4	1.183	0	No
Temperature, °C	-11.1	17.1	1.226	0	Yes
SPO_2, %	-1.2	5.7	1.203	0	No
SOFA	-10.3	-2.3	2.02	1	Pending
SAPS II	-39	8.2	1.761	0	Yes
APS III	-21.3	1.6	1.81	0	Yes
Myocardial infarct	-0.4	6.8	1.079	0	No
Congestive heart failure	-2.6	4.9	1.139	0	No
Peripheral vascular disease	0.3	1.1	1.094	0	No

Cerebrovascular disease	0.5	-1.3	1.067	0	No
Dementia	0.8	0.2	1.055	0	No
Chronic pulmonary disease	-1.4	-12.7	1.147	0	Yes
Rheumatic disease	-0.2	-0.1	1.051	0	No
Diabetes	-0.6	14.1	1.122	0	Yes
Renal disease	-1	-0.8	1.211	0	No
Malignant cancer	-0.4	16.4	1.144	0	Yes
Liver disease	-1.8	7.6	1.085	0	No
Hypertension	-1.3	-4.9	1.306	0	No
UO first 24h, L	-0.4	-0.6	1.045	0	No
RRT, n (%)	-0.6	0.6	1.355	0	No
Dopamine	0	-17.1	1.136	0	Yes
Dobutamine	-1.9	-18.2	1.159	0	Yes
Epinephrine	-0.3	-6.8	1.171	0	No
Norepinephrine	-4.6	0.1	1.326	0	No
Phenylephrine	-1.1	-25.4	1.191	0	Yes

Abbreviations: AG, anion gap; UO, urine output; RRT, renal replacement therapy; WBC, white blood cells; BNU, blood urea nitrogen; PO_2, partial oxygen pressure; PCO_2, partial pressure of carbon dioxide; SBP, systolic blood pressure; DBP, diastolic blood pressure; MBP, mean blood pressure; SPO_2, saturation of peripheral oxygen; SOFA, sequential organ failure assessment score; SAPS II, simplified acute physiology score II; APS III, acute physiology score III; VIF, variance inflation factor.

Table S3 Selection of variables for albumin- adjusted AG

Variables	Change percentage in model I	Change percentage in model II	VIF	colinearity	select
Gender, n (%)	0.7	11.8	1.208	0	Yes
Age, years	1	-29.8	1.27	0	Yes
Ethnicity, n (%)	-0.1	-10	1.109	0	Yes
Marital status, n (%)	7.7	-3.1	1.083	0	No
Weight, kg	-9.7	49	1.182	0	Yes
Platelets, 10^9/L	2.4	2.3	1.184	0	No
WBC, 10^9/L	-0.8	0.4	1.12	0	No
Albumin, g/dL	6.2	-157.9	2.106	1	Pending
BNU, mg/dL	-17	-0.5	1.487	0	Yes
Creatinine, mg/dL	-3.7	-0.6	1.89	0	No
Bicarbonate, mEq/L	-28.6	-27.9	6.801	1	Pending
Calcium, mg/dL	0.7	3.1	1.262	0	No
Chloride, mEq/L	6.4	-93.6	7.235	1	Pending
Sodium, mEq/L	-1.4	-117.6	5.358	1	Pending
Potassium, mEq/L	-5.3	38.3	2.462	1	Pending
Lactate, mmol/L	-24.7	-1.2	1.399	0	Yes
Glucose, mmol/L	0.4	-0.2	1.16	0	No
PH	-7.1	18.2	1.419	0	Yes
PO_2	0.5	-31.8	1.283	0	Yes
PCO_2	11.4	-8.4	1.583	0	Yes
Heart rate, beats/min	-2.6	1.8	1.303	0	No
SBP, mmHg	-2.6	-18.8	2.073	1	Pending
DBP, mmHg	2.8	2.5	2.851	1	Pending
MBP, mmHg	2.9	5.6	3.424	1	Pending
Respiratory rate, beats/min	2.7	-0.4	1.183	0	No
Temperature, °C	-12	17.1	1.226	0	Yes
SPO_2, %	-0.5	5.7	1.203	0	No
SOFA	-14.8	-2.3	2.02	1	Pending
SAPS II	-46.3	8.2	1.761	0	Yes
APS III	-31.2	1.6	1.81	0	Yes
Myocardial infarct	-0.3	6.8	1.079	0	No
Congestive heart failure	-2.1	4.9	1.139	0	No
Peripheral vascular disease	0.3	1.1	1.094	0	No

Cerebrovascular disease	0.4	-1.3	1.067	0	No
Dementia	0.6	0.2	1.055	0	No
Chronic pulmonary disease	-1.6	-12.7	1.147	0	Yes
Rheumatic disease	-0.2	-0.1	1.051	0	No
Diabetes	-0.6	14.1	1.122	0	Yes
Renal disease	-1.1	-0.8	1.211	0	No
Malignant cancer	-1.5	16.4	1.144	0	Yes
Liver disease	-2.4	7.6	1.085	0	No
Hypertension	-1.8	-4.9	1.306	0	No
UO first 24h, L	-0.4	-0.6	1.045	0	No
RRT, n (%)	-0.6	0.6	1.355	0	No
Dopamine	0	-17.1	1.136	0	Yes
Dobutamine	-1.8	-18.2	1.159	0	Yes
Epinephrine	-0.4	-6.8	1.171	0	No
Norepinephrine	-6.6	0.1	1.326	0	No
Phenylephrine	-0.6	-25.4	1.191	0	Yes

Abbreviations: AG, anion gap; UO, urine output; RRT, renal replacement therapy; WBC, white blood cells; BNU, blood urea nitrogen; PO_2, partial oxygen pressure; PCO_2, partial pressure of carbon dioxide; SBP, systolic blood pressure; DBP, diastolic blood pressure; MBP, mean blood pressure; SPO_2, saturation of peripheral oxygen; SOFA, sequential organ failure assessment score; SAPS II, simplified acute physiology score II; APS III, acute physiology score III; VIF, variance inflation factor.

Table S4 Subgroup analyses of traditional AG and 28-day ICU mortality in model I

Subgroup	Total	Event (%)	Crude HR (95% CI)	Crude P value	Adj HR (95%CI)	Adj P value	P for interaction
Age, years							
<75	1363	99 (7.3)	1.07 (1.04~1.11)	<0.001	1.09 (1.05~1.13)	<0.001	0.088
⩾75	1927	294 (15.3)	1.04 (1.02~1.06)	<0.001	1.04 (1.02~1.06)	<0.001	
Temperature, °C							
<36.7	1312	181 (13.8)	1.04 (1.02~1.06)	<0.001	1.04 (1.01~1.06)	0.001	0.175
⩾36.7	1978	212 (10.7)	1.07 (1.03~1.1)	<0.001	1.07 (1.04~1.11)	<0.001	
Lactate, mmol/L							
<1.5	1512	129 (8.5)	1.02 (0.99~1.06)	0.209	1.01 (0.98~1.05)	0.467	0.008
⩾1.5	1778	264 (14.8)	1.06 (1.04~1.08)	<0.001	1.07 (1.05~1.1)	<0.001	
SOFA							
<5	1398	58 (4.1)	1.01 (0.95~1.08)	0.716	1.02 (0.95~1.09)	0.638	0.354
⩾5	1892	335 (17.7)	1.05 (1.03~1.07)	<0.001	1.05 (1.03~1.07)	<0.001	
SAPS II							
<38	1619	73 (4.5)	1 (0.95~1.06)	0.976	1 (0.95~1.07)	0.88	0.151
⩾38	1671	320 (19.2)	1.05 (1.03~1.07)	<0.001	1.05 (1.03~1.07)	<0.001	
APS III							
<49	1633	72 (4.4)	1.04 (0.97~1.11)	0.247	1.04 (0.98~1.11)	0.19	0.688
⩾49	1657	321 (19.4)	1.05 (1.03~1.07)	<0.001	1.05 (1.03~1.07)	<0.001	
Dopamine							
No	3116	352 (11.3)	1.05 (1.03~1.07)	<0.001	1.05 (1.03~1.07)	<0.001	0.794
Yes	174	41 (23.6)	1.09 (1~1.18)	0.042	1.07 (0.98~1.17)	0.124	
Dobutamine							
No	3225	362 (11.2)	1.05 (1.03~1.07)	<0.001	1.05 (1.03~1.07)	<0.001	0.233
Yes	65	31 (47.7)	1.06 (0.99~1.13)	0.078	1.09 (1.01~1.17)	0.03	
Phenylephrine							
No	2493	268 (10.8)	1.04 (1.01~1.06)	0.002	1.04 (1.01~1.06)	0.003	0.021
Yes	797	125 (15.7)	1.08 (1.05~1.11)	<0.001	1.09 (1.05~1.12)	<0.001	
RRT							
No	3043	352 (11.6)	1.04 (1.02~1.06)	<0.001	1.04 (1.02~1.06)	<0.001	0.017
Yes	247	41 (16.6)	1.1 (1.04~1.17)	0.001	1.12 (1.05~1.19)	<0.001	
Hypertension							
No	2863	368 (12.9)	1.05 (1.03~1.07)	<0.001	1.05 (1.03~1.07)	<0.001	0.494
Yes	427	25 (5.9)	0.99 (0.88~1.12)	0.876	1 (0.89~1.13)	0.948	

Diabetes							
No	2468	282 (11.4)	1.06 (1.04~1.09)	<0.001	1.08 (1.05~1.1)	<0.001	0.008
Yes	822	111 (13.5)	1.04 (1~1.07)	0.027	1.03 (0.99~1.06)	0.108	
Chronic pulmonary disease							
No	2367	270 (11.4)	1.04 (1.02~1.07)	<0.001	1.04 (1.02~1.06)	<0.001	0.025
Yes	923	123 (13.3)	1.07 (1.04~1.11)	<0.001	1.11 (1.06~1.15)	<0.001	
Renal disease							
No	2302	253 (11)	1.04 (1.02~1.06)	0.001	1.04 (1.01~1.06)	0.002	0.014
Yes	988	140 (14.2)	1.08 (1.05~1.12)	<0.001	1.09 (1.05~1.13)	<0.001	

Abbreviations: SOFA, sequential organ failure assessment score; SAPS II, simplified acute physiology score II; APS III, acute physiology score III; RRT, renal replacement therapy.

Adjusted for gender, age, marital status, ethnicity, weight, albumin, BUN, lactate, PH, PO_2, PCO_2, HR, SBP, RR, temperature, SPO_2, chronic pulmonary disease, renal disease, liver disease, diabetes, hypertension, malignant cancer, dopamine, dobutamine, phenylephrine, RRT, SAPS II, APS III.

Table S5 Subgroup analyses of traditional AG and 28-day ICU mortality in model II

Subgroup	Total	Event (%)	Crude HR (95% CI)	Crude P value	Adj HR (95%CI)	Adj P value	P for interaction
Age, years							
<75	1363	99 (7.3)	1.07 (1.04~1.11)	<0.001	1.06 (1.02~1.11)	0.006	0.325
⩾75	1927	294 (15.3)	1.04 (1.02~1.06)	<0.001	1.02 (1~1.05)	0.114	
Temperature, °C							
<36.7	1312	181 (13.8)	1.04 (1.02~1.06)	<0.001	1 (0.97~1.03)	0.889	0.009
⩾36.7	1978	212 (10.7)	1.07 (1.03~1.1)	<0.001	1.06 (1.02~1.1)	0.003	
Lactate, mmol/L							
<1.5	1512	129 (8.5)	1.02 (0.99~1.06)	0.209	1.01 (0.97~1.05)	0.792	0.379
⩾1.5	1778	264 (14.8)	1.06 (1.04~1.08)	<0.001	1.04 (1.01~1.07)	0.003	
SOFA							
<5	1398	58 (4.1)	1.01 (0.95~1.08)	0.716	1.02 (0.93~1.11)	0.727	0.618
⩾5	1892	335 (17.7)	1.05 (1.03~1.07)	<0.001	1.03 (1~1.05)	0.017	
SAPS II							
<38	1619	73 (4.5)	1 (0.95~1.06)	0.976	0.99 (0.92~1.05)	0.656	0.326
⩾38	1671	320 (19.2)	1.05 (1.03~1.07)	<0.001	1.04 (1.01~1.06)	0.002	
APS III							
<49	1633	72 (4.4)	1.04 (0.97~1.11)	0.247	1.04 (0.97~1.12)	0.291	0.552
⩾49	1657	321 (19.4)	1.05 (1.03~1.07)	<0.001	1.02 (1~1.05)	0.033	
Dopamine							
No	3116	352 (11.3)	1.05 (1.03~1.07)	<0.001	1.02 (1~1.04)	0.041	0.858
Yes	174	41 (23.6)	1.09 (1~1.18)	0.042	1 (0.88~1.14)	0.988	
Dobutamine							
No	3225	362 (11.2)	1.05 (1.03~1.07)	<0.001	1.02 (1~1.05)	0.023	0.563
Yes	65	31 (47.7)	1.06 (0.99~1.13)	0.078	1 (0.88~1.14)	0.995	
Phenylephrine							
No	2493	268 (10.8)	1.04 (1.01~1.06)	0.002	1.02 (0.99~1.05)	0.196	0.501
Yes	797	125 (15.7)	1.08 (1.05~1.11)	<0.001	1.05 (1.01~1.09)	0.009	
RRT							
No	3043	352 (11.6)	1.04 (1.02~1.06)	<0.001	1.02 (1~1.04)	0.087	0.038
Yes	247	41 (16.6)	1.1 (1.04~1.17)	0.001	1.12 (1~1.25)	0.048	
Hypertension							
No	2863	368 (12.9)	1.05 (1.03~1.07)	<0.001	1.02 (1~1.04)	0.033	0.651
Yes	427	25 (5.9)	0.99 (0.88~1.12)	0.876	1.01 (0.86~1.19)	0.907	

Diabetes							
No	2468	282 (11.4)	1.06 (1.04~1.09)	<0.001	1.06 (1.03~1.09)	<0.001	0.006
Yes	822	111 (13.5)	1.04 (1~1.07)	0.027	0.99 (0.95~1.03)	0.715	
Chronic pulmonary disease							
No	2367	270 (11.4)	1.04 (1.02~1.07)	<0.001	1.02 (0.99~1.04)	0.156	0.002
Yes	923	123 (13.3)	1.07 (1.04~1.11)	<0.001	1.12 (1.07~1.17)	<0.001	
Renal disease							
No	2302	253 (11)	1.04 (1.02~1.06)	0.001	1.02 (0.99~1.05)	0.129	0.261
Yes	988	140 (14.2)	1.08 (1.05~1.12)	<0.001	1.05 (1~1.09)	0.043	

Abbreviations: SOFA, sequential organ failure assessment score; SAPS II, simplified acute physiology score II; APS III, acute physiology score III; RRT, renal replacement therapy.

Adjusted for gender, age, marital status, ethnicity, weight, albumin, BUN, lactate, PH, PO_2, PCO_2, HR, SBP, RR, temperature, SPO_2, chronic pulmonary disease, renal disease, liver disease, diabetes, hypertension, malignant cancer, dopamine, dobutamine, phenylephrine, RRT, SAPS II, APS III.

Table S6 Subgroup analyses of albumin-adjusted AG and 28-day ICU mortality in model I

Subgroup	Total	Event (%)	Crude HR (95% CI)	Crude P value	Adj HR (95%CI)	Adj P value	P for interaction
Age, years							
<75	1363	99 (7.3)	1.07 (1.03~1.1)	<0.001	1.08 (1.05~1.12)	<0.001	0.121
⩾75	1927	294 (15.3)	1.04 (1.02~1.06)	<0.001	1.04 (1.02~1.06)	<0.001	
Temperature, °C							
<36.7	1312	181 (13.8)	1.04 (1.02~1.06)	<0.001	1.04 (1.02~1.06)	<0.001	0.239
⩾36.7	1978	212 (10.7)	1.06 (1.02~1.09)	0.001	1.07 (1.03~1.1)	<0.001	
Lactate, mmol/L							
<1.5	1512	129 (8.5)	1.02 (0.98~1.06)	0.291	1.01 (0.98~1.05)	0.481	0.004
⩾1.5	1778	264 (14.8)	1.06 (1.04~1.08)	<0.001	1.07 (1.05~1.1)	<0.001	
SOFA							
<5	1398	58 (4.1)	1.04 (0.99~1.09)	0.149	1.05 (0.99~1.12)	0.081	0.662
⩾5	1892	335 (17.7)	1.05 (1.03~1.06)	<0.001	1.04 (1.03~1.06)	<0.001	
SAPS II							
<38	1619	73 (4.5)	1.01 (0.96~1.06)	0.714	1.02 (0.96~1.08)	0.521	0.401
⩾38	1671	320 (19.2)	1.05 (1.03~1.07)	<0.001	1.05 (1.03~1.07)	<0.001	
APS III							
<49	1633	72 (4.4)	1.08 (1.01~1.14)	0.015	1.08 (1.02~1.15)	0.008	0.228
⩾49	1657	321 (19.4)	1.04 (1.02~1.06)	<0.001	1.04 (1.02~1.06)	<0.001	
Dopamine							
No	3116	352 (11.3)	1.05 (1.03~1.06)	<0.001	1.05 (1.03~1.07)	<0.001	0.894
Yes	174	41 (23.6)	1.06 (0.98~1.16)	0.135	1.05 (0.96~1.14)	0.298	
Dobutamine							
No	3225	362 (11.2)	1.05 (1.03~1.06)	<0.001	1.05 (1.03~1.06)	<0.001	0.485
Yes	65	31 (47.7)	1.05 (0.98~1.12)	0.17	1.07 (0.99~1.15)	0.084	
Phenylephrine							
No	2493	268 (10.8)	1.03 (1.01~1.06)	0.004	1.03 (1.01~1.06)	0.003	0.008
Yes	797	125 (15.7)	1.08 (1.05~1.11)	<0.001	1.09 (1.05~1.12)	<0.001	
RRT							
No	3043	352 (11.6)	1.04 (1.02~1.06)	<0.001	1.04 (1.02~1.06)	<0.001	0.038
Yes	247	41 (16.6)	1.1 (1.03~1.17)	0.002	1.11 (1.04~1.19)	0.001	
Hypertension							
No	2863	368 (12.9)	1.05 (1.03~1.06)	<0.001	1.05 (1.03~1.07)	<0.001	0.571
Yes	427	25 (5.9)	1 (0.9~1.12)	0.956	1.02 (0.91~1.14)	0.717	

Diabetes							
No	2468	282 (11.4)	1.06 (1.03~1.08)	<0.001	1.07 (1.05~1.1)	<0.001	0.008
Yes	822	111 (13.5)	1.04 (1.01~1.07)	0.014	1.03 (1~1.06)	0.065	
Chronic pulmonary disease							
No	2367	270 (11.4)	1.04 (1.02~1.06)	<0.001	1.04 (1.02~1.06)	<0.001	0.062
Yes	923	123 (13.3)	1.06 (1.03~1.1)	<0.001	1.1 (1.05~1.14)	<0.001	
Renal disease							
No	2302	253 (11)	1.04 (1.02~1.06)	0.001	1.04 (1.02~1.06)	0.001	0.027
Yes	988	140 (14.2)	1.08 (1.04~1.11)	<0.001	1.09 (1.05~1.12)	<0.001	

Abbreviations: SOFA, sequential organ failure assessment score; SAPS II, simplified acute physiology score II; APS III, acute physiology score III; RRT, renal replacement therapy.

Adjusted for gender, age, marital status, ethnicity, weight, albumin, BUN, lactate, PH, PO_2, PCO_2, HR, SBP, RR, temperature, SPO_2, chronic pulmonary disease, renal disease, liver disease, diabetes, hypertension, malignant cancer, dopamine, dobutamine, phenylephrine, RRT, SAPS II, APS III.

Table S7 Subgroup analyses of albumin-adjusted AG and 28-day ICU mortality in model II

Subgroup	Total	Event (%)	Crude HR (95% CI)	Crude P value	Adj HR (95%CI)	Adj P value	P for interaction
Age, years							
<75	1363	99 (7.3)	1.07 (1.03~1.1)	<0.001	1.05 (1.01~1.1)	0.017	0.524
⩾75	1927	294 (15.3)	1.04 (1.02~1.06)	<0.001	1.02 (0.99~1.04)	0.182	
Temperature, °C							
<36.7	1312	181 (13.8)	1.04 (1.02~1.06)	<0.001	1 (0.97~1.02)	0.788	0.03
⩾36.7	1978	212 (10.7)	1.06 (1.02~1.09)	0.001	1.06 (1.02~1.1)	0.004	
Lactate, mmol/L							
<1.5	1512	129 (8.5)	1.02 (0.98~1.06)	0.291	1.01 (0.97~1.05)	0.694	0.434
⩾1.5	1778	264 (14.8)	1.06 (1.04~1.08)	<0.001	1.04 (1.01~1.06)	0.007	
SOFA							
<5	1398	58 (4.1)	1.04 (0.99~1.09)	0.149	1.04 (0.96~1.13)	0.289	0.554
⩾5	1892	335 (17.7)	1.05 (1.03~1.06)	<0.001	1.02 (1~1.04)	0.066	
SAPS II							
<38	1619	73 (4.5)	1.01 (0.96~1.06)	0.714	0.99 (0.93~1.06)	0.77	0.554
⩾38	1671	320 (19.2)	1.05 (1.03~1.07)	<0.001	1.03 (1.01~1.05)	0.006	
APS III							
<49	1633	72 (4.4)	1.08 (1.01~1.14)	0.015	1.04 (0.97~1.12)	0.231	0.048
⩾49	1657	321 (19.4)	1.04 (1.02~1.06)	<0.001	1.02 (1~1.04)	0.111	
Dopamine							
No	3116	352 (11.3)	1.05 (1.03~1.06)	<0.001	1.02 (1~1.04)	0.079	0.73
Yes	174	41 (23.6)	1.06 (0.98~1.16)	0.135	0.94 (0.83~1.05)	0.273	
Dobutamine							
No	3225	362 (11.2)	1.05 (1.03~1.06)	<0.001	1.02 (1~1.04)	0.039	0.205
Yes	65	31 (47.7)	1.05 (0.98~1.12)	0.17	0.95 (0.85~1.07)	0.428	
Phenylephrine							
No	2493	268 (10.8)	1.03 (1.01~1.06)	0.004	1.01 (0.99~1.04)	0.299	0.328
Yes	797	125 (15.7)	1.08 (1.05~1.11)	<0.001	1.04 (1~1.08)	0.026	
RRT							
No	3043	352 (11.6)	1.04 (1.02~1.06)	<0.001	1.02 (0.99~1.04)	0.137	0.119
Yes	247	41 (16.6)	1.1 (1.03~1.17)	0.002	1.11 (0.99~1.23)	0.062	
Hypertension							
No	2863	368 (12.9)	1.05 (1.03~1.06)	<0.001	1.02 (1~1.04)	0.077	0.755
Yes	427	25 (5.9)	1 (0.9~1.12)	0.956	1.01 (0.86~1.19)	0.88	

Diabetes							
No	2468	282 (11.4)	1.06 (1.03~1.08)	<0.001	1.05 (1.02~1.08)	<0.001	0.01
Yes	822	111 (13.5)	1.04 (1.01~1.07)	0.014	0.99 (0.95~1.03)	0.57	
Chronic pulmonary disease							
No	2367	270 (11.4)	1.04 (1.02~1.06)	<0.001	1.01 (0.99~1.04)	0.241	0.002
Yes	923	123 (13.3)	1.06 (1.03~1.1)	<0.001	1.11 (1.06~1.16)	<0.001	
Renal disease							
No	2302	253 (11)	1.04 (1.02~1.06)	0.001	1.02 (0.99~1.04)	0.16	0.398
Yes	988	140 (14.2)	1.08 (1.04~1.11)	<0.001	1.04 (0.99~1.08)	0.09	

Abbreviations: SOFA, sequential organ failure assessment score; SAPS II, simplified acute physiology score II; APS III, acute physiology score III; RRT, renal replacement therapy.

Adjusted for gender, age, marital status, ethnicity, weight, albumin, BUN, lactate, PH, PO_2, PCO_2, HR, SBP, RR, temperature, SPO_2, chronic pulmonary disease, renal disease, liver disease, diabetes, hypertension, malignant cancer, dopamine, dobutamine, phenylephrine, RRT, SAPS II, APS III.
